# *Operando* sulfur speciation during sulfur poisoning-regeneration of Ru/SiO_2_ and Ru/Al_2_O_3_ using non-resonant sulfur Kα_1,2_ emission†

**DOI:** 10.1039/d0ra03068f

**Published:** 2020-04-21

**Authors:** Dzulija Kuzmenko, Adam H. Clark, Tilman Schildhauer, Jakub Szlachetko, Maarten Nachtegaal

**Affiliations:** Paul Scherrer Institut (PSI) CH-5232 Villigen Switzerland maarten.nachtegaal@psi.ch; Department of Chemistry and Applied Biosciences, ETH Zürich Vladimir-Prelog-Weg 1-5 8093 Zürich Switzerland; Institute of Nuclear Physics, Polish Academy of Sciences 31-342 Krakow Poland jakub.szlachetko@ifj.edu.pl

## Abstract

A periodic oxidative regeneration of a sulfur-poisoned methanation catalyst is an alternative to the expensive state-of-the-art process of syngas cleaning using wet scrubbers. Here we have employed *operando* X-ray emission spectroscopy (XES) to study sulfur speciation on Ru/SiO_2_ and Ru/Al_2_O_3_ during methanation in the presence of H_2_S and subsequent regeneration in dilute O_2_ at 360 °C. XES allowed us to obtain semi-quantitative sulfur speciation and to monitor changes in the absolute sulfur concentration. It was established that Al_2_O_3_, in contrast to SiO_2_, forms sulfite/sulfate species by reacting with SO_2_, which is released from the poisoned Ru surface upon oxidative treatment. The concentration of sulfite/sulfate species is reduced upon switching the feed to H_2_/CO while no simultaneous increase in sulfide concentration is observed. For both catalysts, the regenerative treatment removes adsorbed sulfur as SO_2_ only partially, which we propose is the main reason for the incomplete activity recovery of the poisoned catalyst after regeneration.

## Abstract 2

*Operando* S Kα X-ray emission spectroscopy allows for a quantitative understanding of the sulfur poisoning and regeneration mechanism of state-of-the-art methanation catalysts used for the wood to synthetic natural gas process.

## Graphical abstract

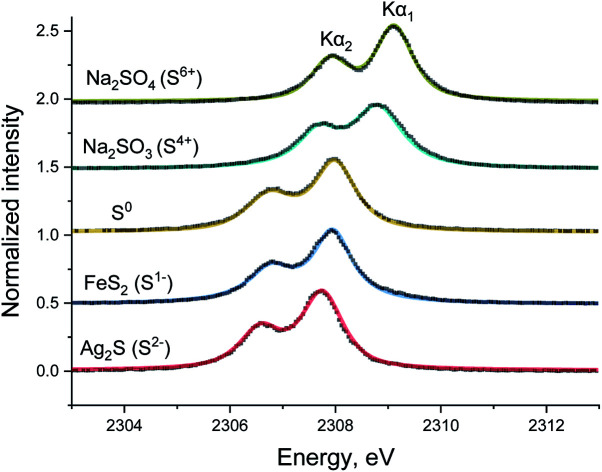

## 1. Introduction

The production of methane from dry biomass consists of four main process steps: biomass gasification, syngas cleaning to remove catalyst poisons such as sulfur-containing compounds (H_2_S, COS, and thiophene), methanation (CO + 3H_2_ ⇌ CH_4_ + H_2_O) and removal of H_2_O and CO_2_. The second step, syngas cleaning, is currently energy inefficient since it requires cooling of syngas to ambient temperature between the gasification and methanation steps, which are performed at around 850 °C and 400 °C respectively.^1,2^ We have been exploring an alternative process, where the low temperature syngas cleaning step is skipped and the methanation catalyst is periodically regenerated after sulfur poisoning which would make the whole dry biomass to methane process more energy efficient.^3^

We have reported recently that the ability to regenerate sulfur poisoned ruthenium nanoparticles, as measured by its methanation activity, is improved when ruthenium nanoparticles are supported on a sulfur-inert support, SiO_2_ compared to Al_2_O_3_.^4^ This was attributed to a higher Ru dispersion and lower sulfur storage of SiO_2_ upon multiple poisoning-regeneration cycles. With the help of *in situ* diffuse reflectance infrared spectroscopy, we were able to monitor the speciation of some of the sulfur species, where the formation of sulfate and sulfite species was observed when the poisoned sulfided catalyst was exposed to dilute oxygen. During subsequent exposure to methanation conditions (H_2_/CO) the intensity of the sulfate and sulfite peak decreased, but we could not determine if this was related to a reduction of sulfate and sulfite species to gas phase SO_2_ or to the sulfate reduction and subsequent adsorption of sulfides since diffuse reflectance spectroscopy did not allow detecting sulfides. Another open question is how efficient the regeneration process is, *i.e.* what percentage of initially adsorbed sulfur is removed from the catalyst surface *via* oxidative treatment. To answer these questions, it would be valuable to find ways to quantitatively trace sulfur speciation under *operando* conditions.

In that context, X-ray spectroscopy would be a possible solution since it allows for detecting all sulfur species. Our group has recently reported a study on the mechanism of sulfur poisoning on Ru/Al_2_O_3_ by using *in situ* sulfur K-edge X-ray absorption (XAS),^5^ where we proposed that sulfate species, formed during oxidative regeneration, under subsequent reducing conditions convert to H_2_S, which re-poisons Ru nanoparticles. However, S K-edge XAS only provided qualitative information. On the other hand, semi-quantitatively sulfur speciation under *operando* conditions should be accessible by sulfur X-ray emission spectroscopy (XES). Indeed, while XAS probes the unoccupied states and has many overlapping features, reflecting not only the sulfur oxidation state but also the local coordination of the sulfur atom,^6^ XES gives information on the occupied states and primarily reflects the nuclear charge. The sulfur Kα_1,2_ emission signal appears in the XES spectrum as a doublet, which can be readily fitted with a convolution of two pseudo-Voigt function peaks. For different sulfur oxidation states, only a systematic shift in peak energy position and a slight change in the ratio of Kα_1_ to Kα_2_ intensity have been observed.^7^ This allows obtaining quantitative sulfur speciation from XES using, for example, a linear combination fitting approach, quick and straightforward. In addition, since the X-ray emission signal intensity is directly proportional to the number of sulfur atoms in the beam, the changes in signal intensity can be used to estimate the changes in sulfur concentration. We have recently reported the development of a dedicated non-resonant XES setup including *operando* cell that allows to study sulfur speciation at low concentrations and in a time-resolved manner.^8^ Here, by employing non-resonant sulfur X-ray emission spectroscopy, we investigate the poisoning and regeneration mechanisms of sulfur-poisoned Ru nanoparticles supported on SiO_2_ and Al_2_O_3_. The key difference between the two catalysts arises from the ability of Al_2_O_3_ to form sulfates by reacting with SO_2_, which is released from the Ru surface upon oxidative treatment. By using *operando* non-resonant S XES, we show that for both catalysts, oxidative treatment at 360 °C only partially removes adsorbed sulfur as SO_2._ Sulfur remains adsorbed on the metal surface as sulfite/sulfate species. When the oxygen supply is removed, these oxidized species are reduced back to sulfide, which is the reason for the incomplete activity recovery.

## 2. Experimental

Non-resonant sulfur XES measurements were carried out using a dedicated S XES von Hamos spectrometer including an *operando* spectroscopic cell^8^ installed at the SuperXAS beamline of the Swiss Light Source (SLS), Villigen, Switzerland. The SLS operates at 400 mA and 2.4 GeV. The polychromatic beam from a 2.9 Tesla superbend magnet was collimated by a Si-coated mirror at 2.5 mrad and subsequently monochromatized by a Si (111) channel-cut crystal. Downstream of the monochromator, a Rh-coated toroidal mirror was used to focus the incident X-rays to a spot size of 100 × 100 μm at the sample position. The incoming X-ray energy was calibrated by using an Fe foil in transmission mode at the start of the experiment. The dedicated XES spectrometer^9^ was enclosed in a stainless steel chamber evacuated by a turbo pump to 10^−3^ to 10^−4^ mbar. In this vacuum chamber a 15 cm bending radius Si (111) von Hamos crystal was placed on a motorized stage allowing to fine tune the crystal position in focusing direction. A charge-coupled device (CCD) detector (Andor DO440, 2048 × 512 array with 13.5 × 13.5 μm pixels), which was thermoelectrically cooled to −30 °C, was used as a position sensitive detector. The chip length, consisting of 2048 pixels, allows measuring the X-ray emission energy range from 2.255 to 2.348 keV in a single acquisition. An aluminized 6 μm thick Mylar window was placed in front of the CCD chip to prevent visible light from reaching the chip. According to Bragg's law, the center of the Si (111) von Hamos crystal diffracts not only the Kα emission but also triple the energy of the sulfur Kα emission, *i.e.* 6.921 keV. Hence, the incident energy for non-resonant XES was set to be around 6.9 keV since this allowed us to observe both sulfur Kα emission and elastic scattering of the incident 6.9 keV beam in the same spectrum. The elastic scattering peak position was subsequently used for CCD pixel to energy calibration.

For performing *operando* experiments, the *operando* cell^9^ was filled with *ca.* 15 mg of 3% Ru/SiO_2_ and Ru/Al_2_O_3_, prepared according to ref. 4. The *operando* cell is operated at atmospheric pressure and is equipped with a thermocouple close to the catalyst bed. A 7 μm thin Kapton film was used as window material. The cell was heated to 360 °C (10 °C min^−1^ ramp) while He was flowing through the cell. Subsequently, the catalyst was treated with the following gas sequence: methanation in the presence of inorganic sulfur (0.5 vol% CO, 2.5 vol% H_2_, 100 ppm H_2_S), He flush, oxidative regeneration (1 vol% O_2_), He flush and methanation without sulfur poisons (0.5 vol% CO, 2.5 vol% H_2_). The gases were dosed with mass flow controllers (Bronkhorst) and the gas phase after the reactor cell was analyzed with a quadrupole mass spectrometer (Max 300-LG, Extrel).

Reference compounds (Ag_2_S, FeS_2_, S, Na_2_SO_3_ and Na_2_SO_4_) were purchased from Sigma Aldrich and used as received. The powders were crushed using a pestle and mortar and subsequently pressed in a 0.5 cm diameter pellet without addition of any binder. Emission spectra were recorded for 60 seconds, apart for the elemental S sample, where an acquisition time of 5 seconds was sufficient to obtain a high signal-to-noise ratio.

The raw spectra were baseline corrected and normalised by the total area under the peak using an in-house written Python script. The normalised spectra of the reference compounds were used for fitting *operando* spectra. The following procedure for fitting the reference non-resonant XES spectra was followed: pseudo-Voigt peak functions^10^ were fitted to Kα_1_ and Kα_2_ emission peaks of the reference compounds and the extracted values are peak center, full width at half maximum (FWHM). The peak profile of the reference compounds was used for fitting the unknown *operando* spectra. The data treatment was performed using Origin software.

## 3. Results and discussion

### 3.1. Reference sulfur compounds

**Fig. 1:**
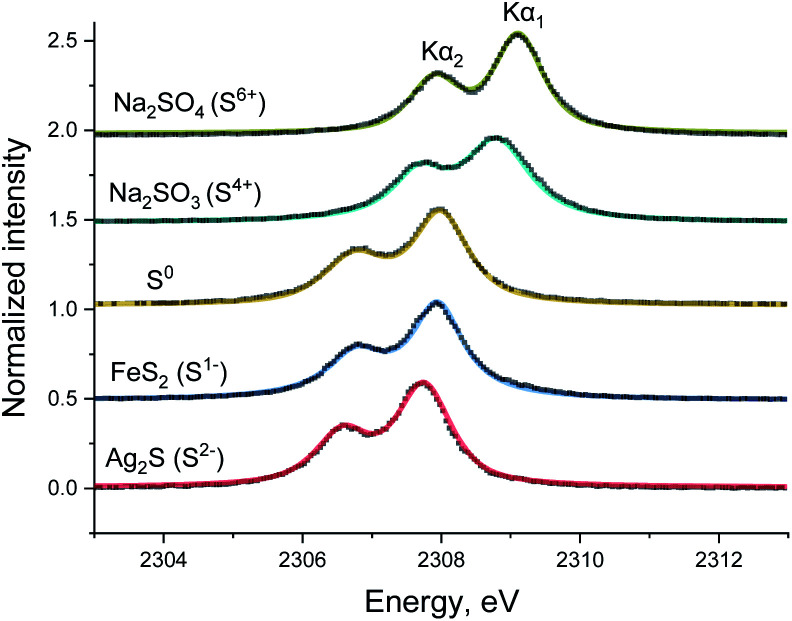
Sulfur Kα_1,2_ emission spectra (dotted black line) and the corresponding pseudo-Voigt peak function fit (colored full line) of reference compounds: Ag_2_S, FeS_2_, S, Na_2_SO_3_, and Na_2_SO_4_.^9^

The spectra of various sulfur reference compounds Ag_2_S (S^2−^), FeS_2_ (S^1−^), S (S^0^), Na_2_SO_3_ (S^4+^) and Na_2_SO_4_ (S^6+^) were previously measured by our group^8^ and the published spectra are shown in Fig. 1 to help the readers with *in situ* spectra interpretation. In these spectra, the Kα_1_ and Kα_2_ lines can be clearly resolved, and an energy shift for various sulfur oxidation states is also observed, similar to previously reported values.^7,11,12^ Table S1 in ESI† gives the parameters (peak center, FWHM *etc.*) extracted from fitting the reference and used for fitting of the *operando* spectra described below.

### 3.2. Ru/SiO_2_

**Fig. 2:**
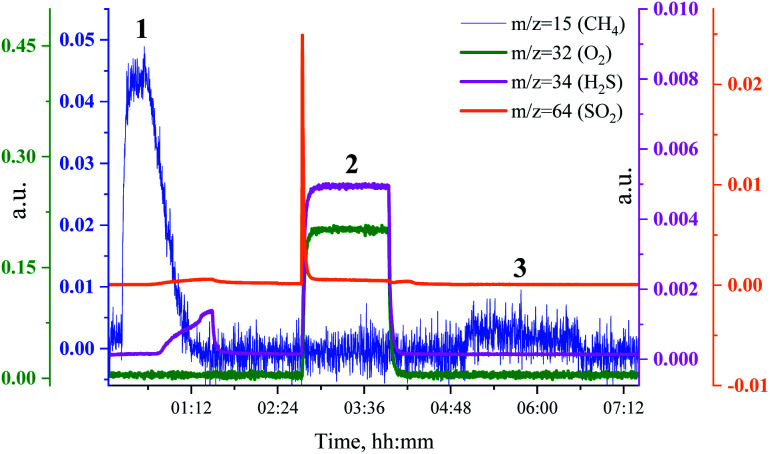
MS traces for CH_4_ (*m*/*z* = 15, blue), O_2_ (*m*/*z* = 32, green and *m*/*z* = 34, purple), H_2_S (*m*/*z* = 34, purple) and SO_2_ (*m*/*z* = 64, orange) during poisoning-regeneration of Ru/SiO_2_ at 360 °C. H_2_S breakthrough is observed.

To investigate the relationship between the catalyst methanation activity and its structure during sulfur poisoning and regeneration, we have carried out H_2_S poisoning, oxidative regeneration and subsequent sulfur-free methanation of Ru/SiO_2_ while simultaneously measuring non-resonant X-ray emission spectra. Fig. 2 shows the mass spectrometer (MS) traces for CH_4_ (*m*/*z* = 15), O_2_ (*m*/*z* = 32 and *m*/*z* = 34), H_2_S (*m*/*z* = 34) and SO_2_ (*m*/*z* = 64) recorded during poisoning (H_2_/CO/H_2_S labelled 1), regeneration (O_2_ labelled 2) and subsequent sulfur-free methanation (H_2_/CO labelled 3) of Ru/SiO_2_. Note that the increase in *m*/*z* = 34, that accompanies the increase in *m*/*z* = 32 during oxidation, is solely due to oxygen, because the O atom has two important isotopes: ^16^O and ^18^O and hence the O_2_ molecule produces *m*/*z* = 32 and *m*/*z* = 34 fragments. The initial level of methane production, when Ru/SiO_2_ is treated with H_2_/CO/H_2_S, decreases gradually because of H_2_S adsorption on the active sites of Ru nanoparticles. The breakthrough of H_2_S is observed as the catalyst loses its methanation activity (*m*/*z* = 34 in Fig. 2). After methanation activity dropped to zero, the catalyst is treated with dilute oxygen to remove adsorbed sulfur atoms as SO_2_, which was detected as a spike in the MS data (*m*/*z* = 64 in Fig. 2). In the subsequent sulfur-free H_2_/CO treatment, we see a small recovery of methanation activity (*m*/*z* = 15 in Fig. 2). The recovered methanation activity is smaller than that reported in our previous studies^4^ probably due to the lower regeneration temperature (360 °C *vs.* 430 °C) used in this *operando* experiment.

**Fig. 3:**
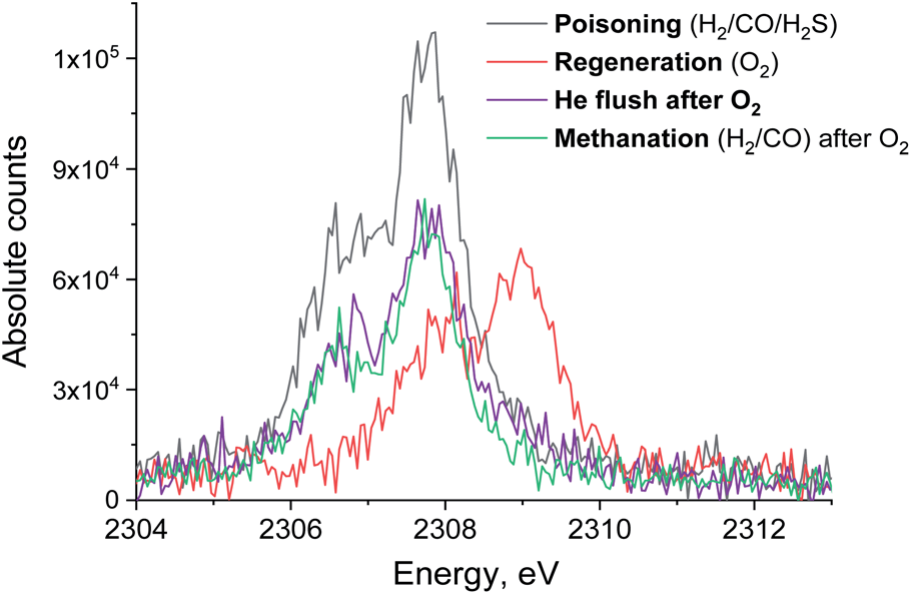
S Kα XES spectra of Ru/SiO_2_ in H_2_/CO/H_2_S (black), O_2_ (red) and subsequent pure He (purple) and H_2_/CO atmospheres (green). Integration time for each spectrum is 13 minutes.

Fig. 3 shows the non-resonant XES spectra for all stages of the sulfur poisoning-regeneration cycle for methanation over a Ru/SiO_2_ catalyst. First, we observe a peak corresponding to reduced sulfur species (Fig. 3, black), which likely results from dissociative adsorption of H_2_S on the ruthenium surface since SiO_2_ is not known to form sulfides. *In situ* DRIFTS is not able to detect this reduced species^4^ likely because of the low IR intensity of this species and/or their vibration frequency being beyond the detectable range.^13^ Upon exposure to dilute oxygen, we see that the emission peak shifts to higher energy and its intensity decreases by *ca.* a factor of two (Fig. 3, red). The intensity decrease suggests that around half of the sulfidic species leave the metal surface as gas phase SO_2_, consistent with the observation of a spike of SO_2_ in the MS trace signal (Fig. 2, orange). The remaining oxidized species could be located on the support, SiO_2_, or on the Ru nanoparticles. We have previously shown^4^ using *in situ* DRIFTS that sulfur species were only observed upon treatment of sulfur poisoned Ru/SiO_2_ as a small peak at 1425 cm^−1^ (which was assigned to sulfate species on SiO_2_ (ref. 14–17)) when the O_2_ concentration is increased from 1 to 5% (Fig. S1†). Importantly, due to absorption of IR radiation below 1250 cm^−1^ by bulk Si–O bonds, we were only able to examine the region above 1250 cm^−1^ for *in situ* DRIFTS experiment. This implies that all species appearing below 1250 cm^−1^ will be invisible. This could have potentially masked sulfate species, as they could appear below 1250 cm^−1^,^18^ formed when sulfur poisoned Ru/SiO_2_ was treated with 1% O_2_ in Ar.

When the flow of oxygen is switched to pure He, the non-resonant XES spectra show that S^4+^/S^6+^ species are fully converted to S^2−^ (Fig. 3, purple), indicating that S^4+^/S^6+^ species are not stable in the absence of oxygen. No significant changes to the XES spectrum are observed when CO and H_2_ are introduced in the feed (Fig. 3, green). We propose that the sulfidic species present after the oxidative regeneration (and flushing with inert gas) are blocking the active sites for methanation and cause the loss of Ru/SiO_2_ methanation activity. We also observe that when the gas feed is switched to O_2_, the intensity of the emission peak decreases by half, indicating that only half of the sulfur species at the position where the X-ray beam impinges the sample desorb as SO_2_. This means that at the temperature of this experiment, 360 °C, only half of the poisoning species are removed from the surface. Increasing the temperature of the oxidative treatment could improve the regeneration efficiency because more sulfur atoms could desorb as SO_2_.

### 3.3. Ru/Al_2_O_3_

**Fig. 4:**
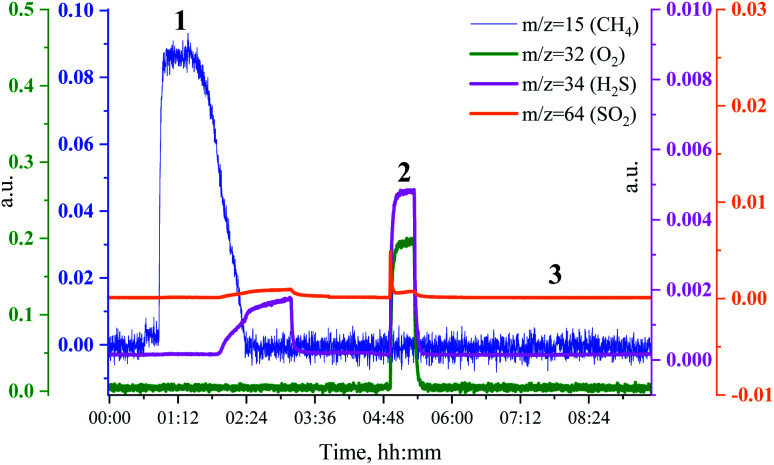
MS trace for CH_4_ (*m*/*z* = 15, blue), O_2_ (*m*/*z* = 32, green and *m*/*z* = 34, purple), H_2_S (*m*/*z* = 34, purple) and SO_2_ (*m*/*z* = 64, orange) during poisoning-regeneration of Ru/Al_2_O_3_ at 360 °C. H_2_S breakthrough is observed at 1 h 15 min.

To see if the methanation activity of the Ru/Al_2_O_3_ catalyst recovers after sulfur poisoning, we have carried out H_2_S poisoning, oxidative regeneration and subsequent sulfur-free methanation of Ru/Al_2_O_3_. In addition, non-resonant X-ray emission spectra were acquired simultaneously at each stage. Fig. 4 shows the MS traces for CH_4_ (*m*/*z* = 15), O_2_ (*m*/*z* = 32, *m*/*z* = 34), H_2_S (*m*/*z* = 34) and SO_2_ (*m*/*z* = 64) recorded during poisoning (H_2_/CO/H_2_S labelled 1), regeneration (O_2_ labelled 2) and subsequent sulfur-free methanation (H_2_/CO labelled 3) over a Ru/Al_2_O_3_ catalyst. When treated with H_2_/CO/H_2_S, Ru/Al_2_O_3_ also experienced methanation activity loss. However, deactivation of Ru/Al_2_O_3_ (and for H_2_S to break through) is *ca.* twice slower than observed for Ru/SiO_2_ (*cf.*Fig. 2 and 4). As we have previously proposed,^4^ this could result from two causes. First, since Ru/Al_2_O_3_ has a smaller initial particle size than Ru/SiO_2_, there are more active sites and hence the deactivation is slower. Secondly, H_2_S adsorbs on to the Al_2_O_3_ surface, which also slows down deactivation of Ru nanoparticles. After regeneration however, no methanation activity is observed probably because of the long H_2_S treatment resulting in a high sulfur coverage that made oxygen adsorption on to the poisoned surface difficult or due to a low regeneration temperature. In addition, the SO_2_ signal during oxidative regeneration for Ru/Al_2_O_3_ is much smaller than for Ru/SiO_2_ (*cf.*Fig. 2 and 4, the scale for the SO_2_ signal is kept the same in Fig. 2 and 4 to simplify comparison), suggesting that the Al_2_O_3_ support reacts with SO_2_ released from Ru nanoparticles upon oxidative regeneration.

**Fig. 5:**
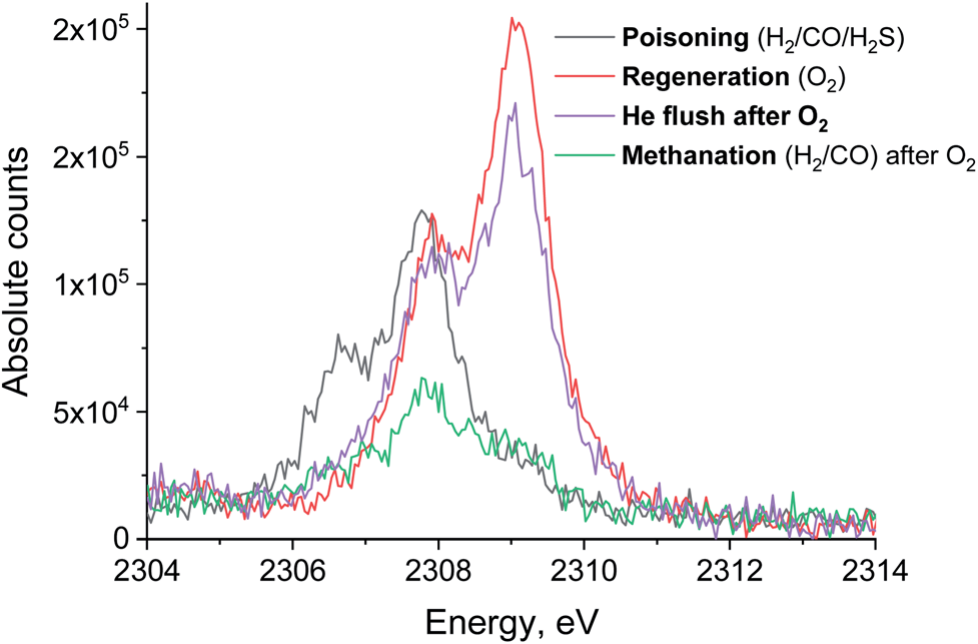
S XES spectrum of Ru/Al_2_O_3_ in H_2_/CO/H_2_S (black), O_2_ (red) and subsequent pure He (purple) and H_2_/CO atmosphere (green). Integration time for each spectrum is 13 minutes.

**Fig. 6:**
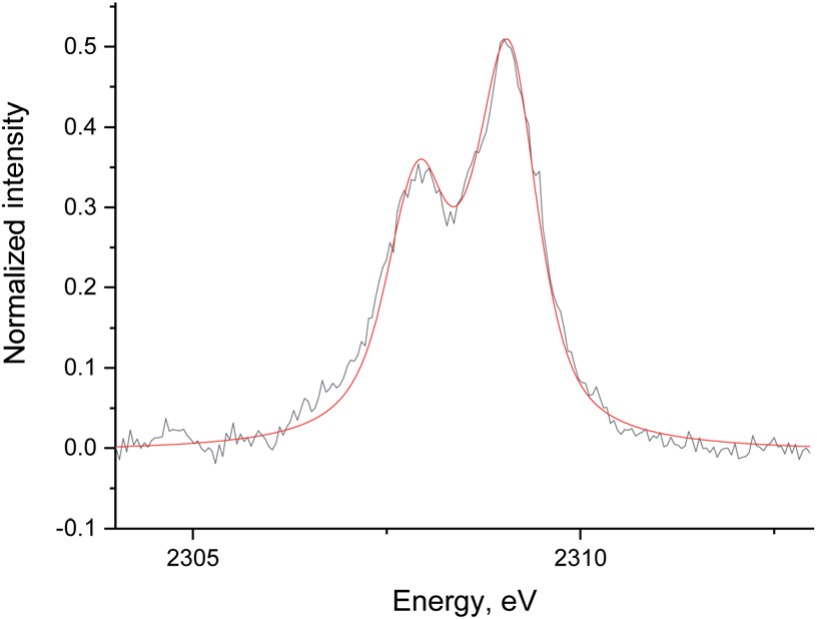
The data (black) and fit (red) of the Ru/Al_2_O_3_ S XES spectrum in He without S^2−^ reference added. The data was background corrected and normalized by integral intensity.

**Fig. 7:**
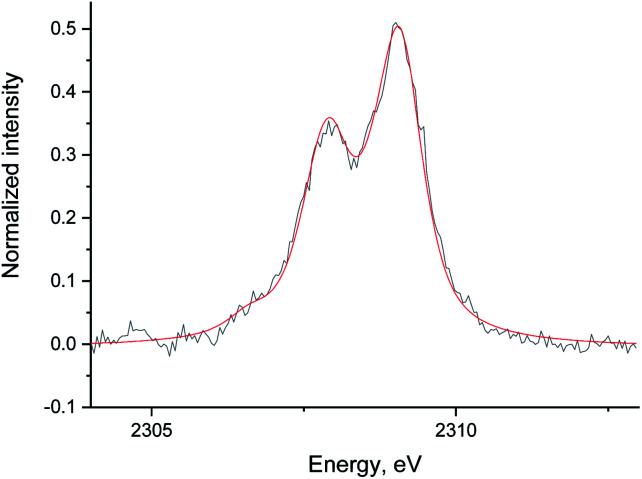
The data (black) and fit (red) of the Ru/Al_2_O_3_ S XES spectrum in He with S^2−^ reference added. The data was background corrected and normalized by integral intensity.

**Fig. 8:**
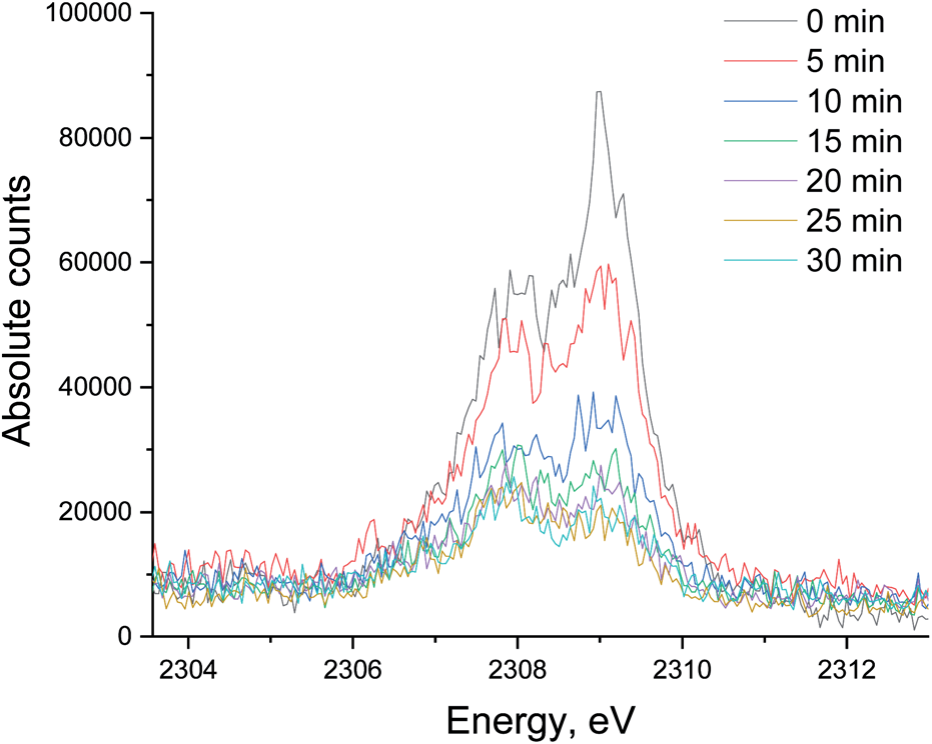
The evolution of the sulfur XES signal during H_2_/CO treatment after oxidative regeneration of Ru/Al_2_O_3_. Integration time for each spectrum is 5 minutes.

**Fig. 9:**
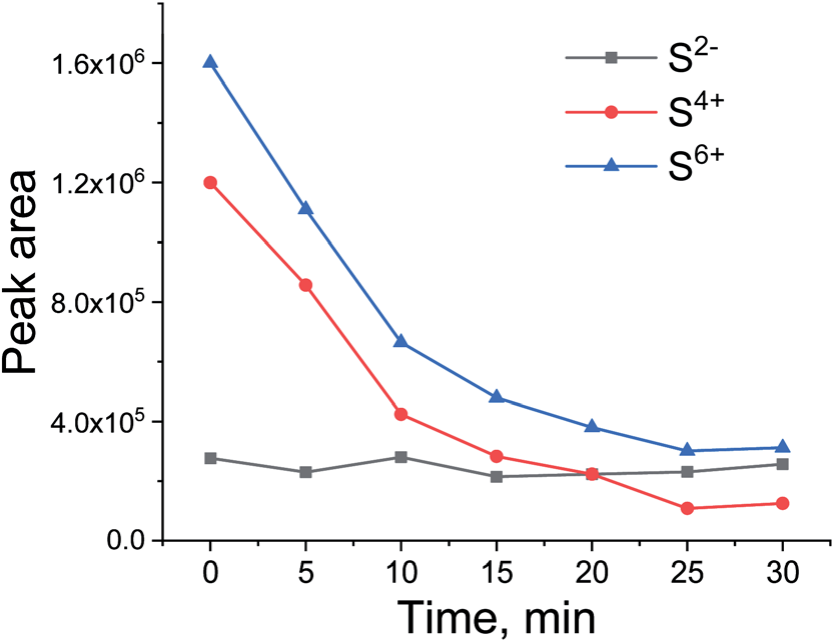
Peak area of S^2−^, S^4+^, S^6+^ during H_2_/CO treatment after oxidative regeneration of Ru/Al_2_O_3_.

Fig. 5 shows the non-resonant XES spectra for all stages of the sulfur poisoning-regeneration cycle for Ru/Al_2_O_3_. As for Ru/SiO_2_, after H_2_/CO/H_2_S treatment we observe a spectrum, which corresponds to sulfidic species (Fig. 5, black). When the poisoned catalyst is contacted with oxygen, SO_2_ is removed from the surface of the metal and reacts with Al_2_O_3_, forming S^4+^ and S^6+^ species. When looking at raw counts (Fig. 5, *cf.* black and red), we observe that the signal intensity of these oxidized species is higher than the intensity of the sulfide. The observed signal intensity change can be explained by the fact that the X-rays are probing the middle of the reactor: SO_2_ desorbed from the metal surface at the inlet of the reactor reacts with Al_2_O_3_ in the middle (also probably the outlet of the reactor) increasing the overall signal intensity. When the flow is switched from 1% O_2_ to pure He (Fig. 5, *cf.* red and purple), we observe the appearance of a small shoulder at lower energies. Fig. 6 shows the fit of this spectrum with a linear combination of S^4+^ and S^6+^ references. When adding 10% of the S^2−^ reference to the linear combination of S^4+^ and S^6+^ references, the fit also accounts for the low energy shoulder-like feature as is shown in Fig. 7. This can be explained by the presence of two different oxidized species – one on the surface of the metal and another one on the support. The former as in the case of Ru/SiO_2_ (Fig. 3) are converted to S^2−^ when the oxygen supply is switched off, while the latter, presumably, in the form of Al_2_(SO_4_)_3_, are stable even without oxygen in the feed. After 30 minutes of subsequent H_2_/CO treatment (Fig. 5, green) a decrease of the sulfate signal intensity is observed. Fig. 8 shows the sulphur XES signal temporal evolution during H_2_/CO treatment and Fig. 9 shows the peak area of each species every 5 minutes derived from fitting (Fig. S2–S7 in ESI†) of the spectra using S^2−^, S^4+^, S^6+^ references. The fitting parameters of the references and the percentage of each reference component in the fit of the *in situ* spectra are given in Tables S1 and S2† respectively. From Fig. 9, we can conclude that the absolute concentration of S^4+^ and S^6+^ is decreasing while the concentration of S^2−^ stays constant during H_2_/CO treatment.

**Fig. 10:**
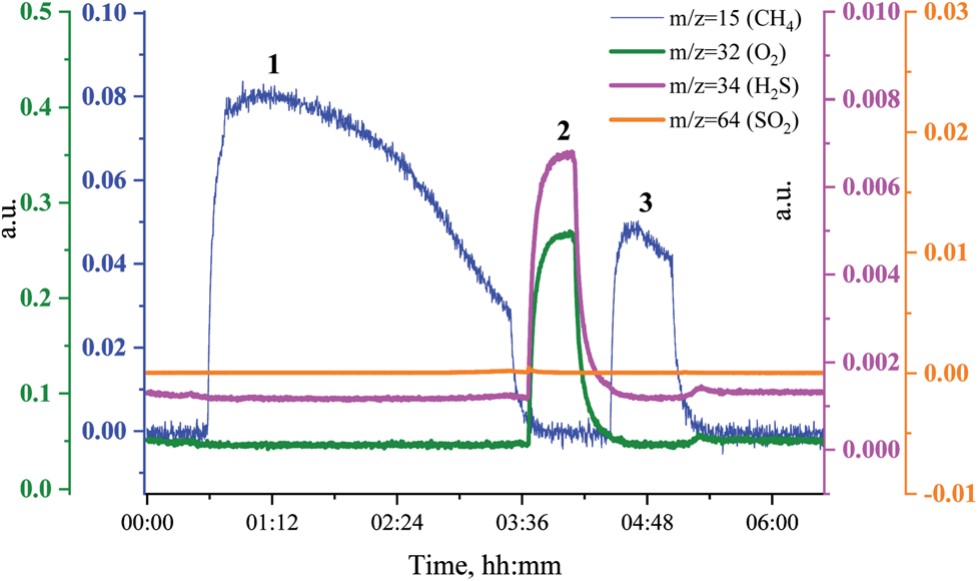
MS traces for CH_4_ (*m*/*z* = 15, blue), O_2_ (*m*/*z* = 32, green and *m*/*z* = 34, purple), H_2_S (*m*/*z* = 34, purple) and SO_2_ (*m*/*z* = 64, orange) during poisoning-regeneration of Ru/Al_2_O_3_ at 360 °C. No H_2_S breakthrough is observed.

In a separate experiment, Ru/Al_2_O_3_ was exposed to H_2_/CO/H_2_S at the same temperature and gas flow for a shorter period, which resulted in an incomplete deactivation and methanation activity being observed in the MS traces after oxidative treatment. Fig. 10 shows the MS traces for CH_4_ (*m*/*z* = 15), O_2_ (*m*/*z* = 32 and *m*/*z* = 34), H_2_S (*m*/*z* = 34) and SO_2_ (*m*/*z* = 64) recorded during poisoning (H_2_/CO/H_2_S labelled 1), regeneration (O_2_ labelled 2) and sulfur-free methanation (H_2_/CO labelled 3). The methanation activity is not fully lost due to poisoning and, importantly, no H_2_S breakthrough is seen (Fig. 10, pink). Further, no or only a very small SO_2_ peak is visible upon O_2_ addition.

**Fig. 11:**
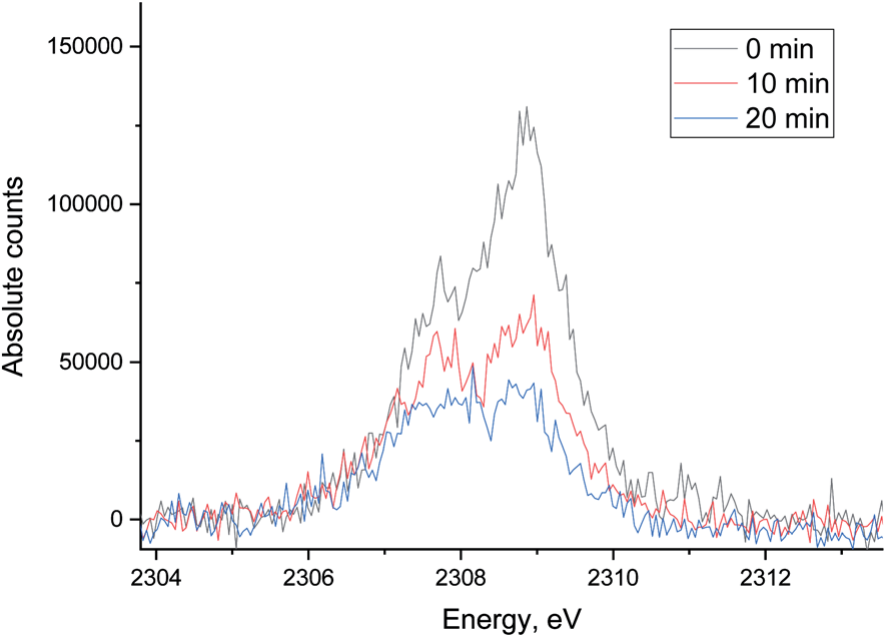
The evolution of sulfur XES signal during H_2_/CO treatment after oxidative regeneration of Ru/Al_2_O_3_. Integration time for each spectrum is 10 minutes.

**Fig. 12:**
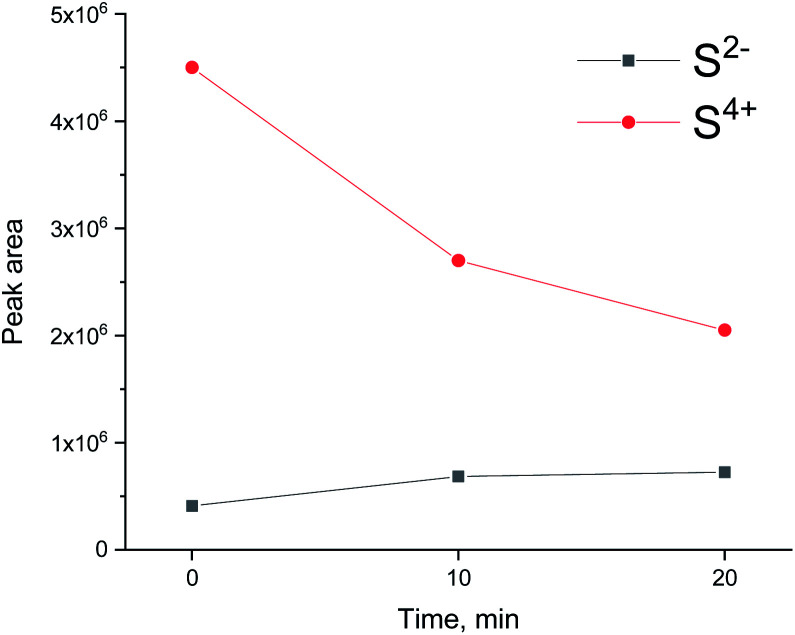
Peak area of S^2−^, S^4+^, S^6+^ during H_2_/CO treatment after oxidative regeneration of Ru/Al_2_O_3_.

The evolution of XES spectra during H_2_/CO treatment is shown in Fig. 11 and the peak area for each species is shown in Fig. 12 (fitting in S8–S10 in ESI†). Overall, we observe a decrease in the total area of the peak as H_2_/CO treatment proceeds, the intensity of the oxidized species (S^4+^ only could be fitted) peak decreases and there is a small increase in the peak area of S^2−^. This suggests that there is possibly a small chance of a partial sulfite reduction to sulfide for a partially poisoned catalyst. Most of the oxidized species however are reduced to gas phase SO_2_ rather than to adsorbed sulfidic species.

### 3.4. Proposed mechanism

**Fig. 13:**
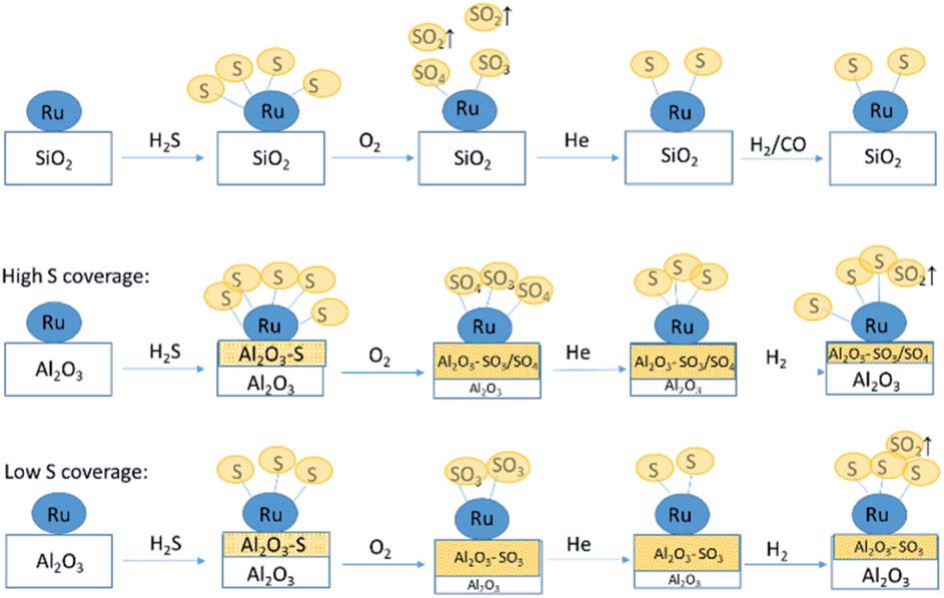
Proposed mechanism of sulfur poisoning and regeneration of Ru/SiO_2_ (top) and Ru/Al_2_O_3_ (middle and bottom).

By combining all results presented here, we propose the following mechanisms of sulfur transport on Ru/SiO_2_ and Ru/Al_2_O_3_ (Fig. 13). On Ru/SiO_2_, around half of the S^2−^/S^1−^ species, formed on the surface of the Ru metal during poisoning *via* dissociative adsorption of H_2_S, are oxidized upon oxygen introduction at 360 °C and released as gas phase SO_2_ while the other half remains bound to the metal surface as a mixture of S^4+^/S^6+^ species. As oxygen is removed from the feed, S^4+^/S^6+^ species on the Ru nanoparticle surface are reduced back to S^2−^/S^1−^ and subsequent H_2_/CO treatment does not change the speciation significantly. The main difference for Ru/Al_2_O_3_ is that oxidation also produces S^4+^/S^6+^ species bound to the support, Al_2_O_3_, which are stable when oxygen is removed from the feed and are partially removed (likely reduced to gas phase species) when H_2_/CO is introduced. In addition, for Ru/Al_2_O_3_, the mechanism also depends on the sulfur surface coverage established during H_2_S poisoning. For high coverage, no increase in S^2−^ species concentration accompanies S^4+^/S^6+^ partial removal, while for low coverage we see a small increase in S^2−^ species as the number of S^4+^/S^6+^ species decreases. This can be explained by the difference in the availability of adsorption sites on Ru: a heavily poisoned catalyst has less sites which are available for sulfide re-adsorption.

## 4. Conclusions

In order to understand the influence of periodic oxidative regeneration of sulfur-poisoned methanation catalysts as alternative to cost intensive low temperature scrubbers, mechanistic studies are indispensable. Based on the previous study of sulfur poisoning-regeneration of a Ru/Al_2_O_3_ catalyst using *operando* sulfur K-edge XAS, the reduction of sulfate species, formed during oxidative regeneration, under subsequent reducing conditions to sulfides was proposed. However, since no exact reference compound spectra were available, only qualitative information was obtained. We have shown here that *operando* non-resonant X-ray emission spectroscopy is an alternative powerful technique for studying sulfur speciation. In contrast to XAS, XES allowed us to monitor the changes in the absolute concentration of various sulfur species during poisoning-regeneration and determine the proportion of each oxidation state species by a straightforward linear combination fitting of the references.

We have established that oxygen treatment of both poisoned catalysts removes adsorbed sulfur as SO_2,_ but only partially. The remaining part stays adsorbed on to Ru nanoparticles as S^4+^/S^6+^ species. These species are only stable under oxygen flow and reduce back to sulfide when no oxygen is in the feed. The loss of methanation activity is due to an incomplete removal of sulfur species from the Ru metal surface during oxidative regeneration and an increase of the oxidative regeneration temperature could help in removing more sulfur species from the surface to the gas phase.

## Conflicts of interest

There are no conflicts to declare.

## Supplementary Material

RA-010-D0RA03068F-s001
