# Supplementary material for: Operando sulfur speciation during sulfur poisoning-regeneration of Ru/SiO2 and Ru/Al2O3 using non-resonant sulfur Kα1,2 emission
Source: RSC Adv. 2020 Apr 21;10(27):15853–9. doi: 10.1039/d0ra03068f (PMC9052376; doi:10.1039/d0ra03068f)
Supplement: RA-010-D0RA03068F-s001 [file RA-010-D0RA03068F-s001.pdf]

Electronic Supplementary Material (ESI) for RSC Advances.
This journal is © The Royal Society of Chemistry 2020

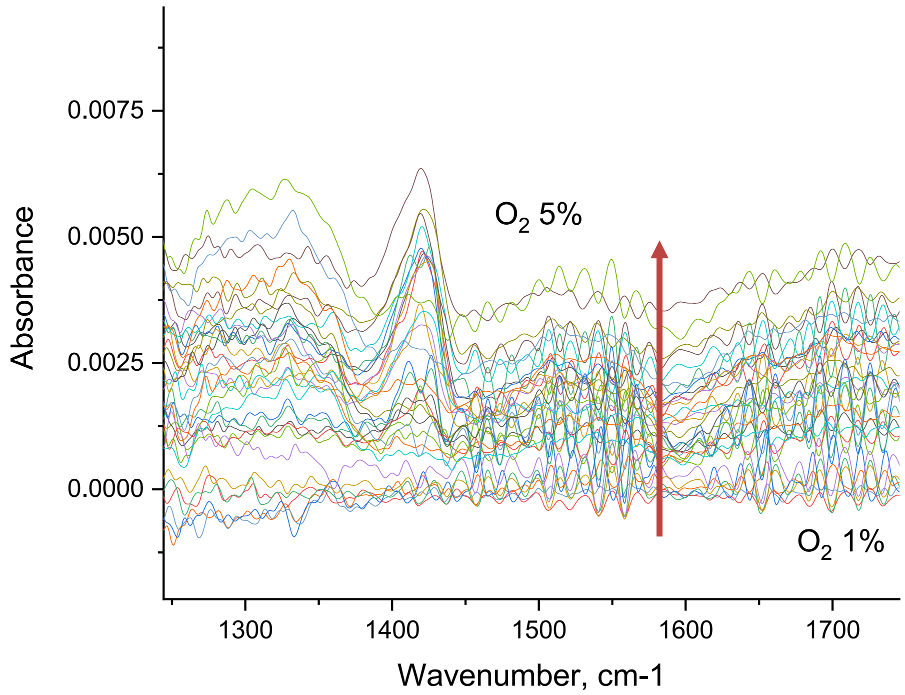

Fig. S1: DRIFTS spectra of poisoned Ru/SiO_2 exposed to oxidative regeneration at increasing oxygen concentration

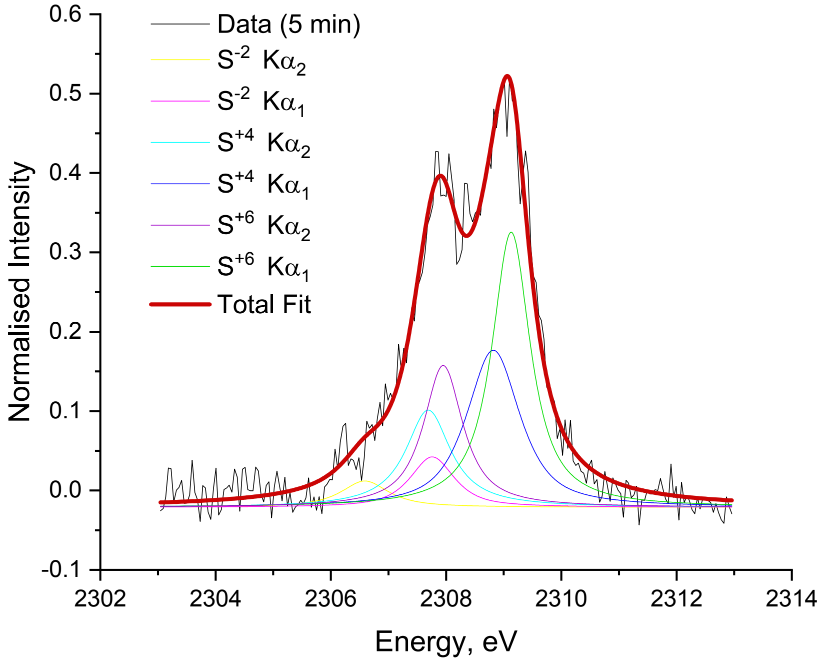

Fig. S2: 5 min into H_2/CO treatment

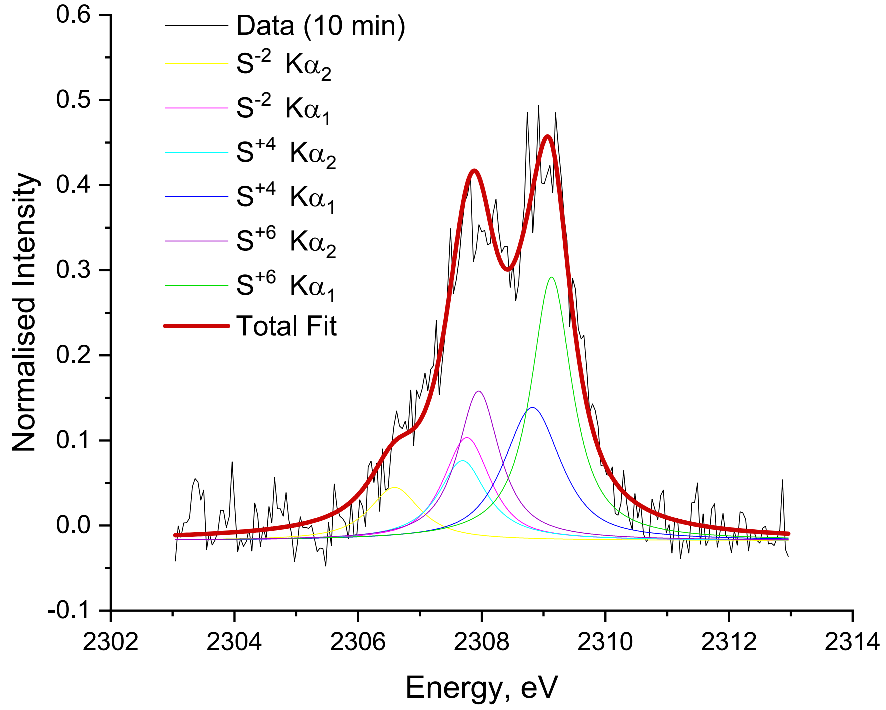

Fig. S3: 10 min into H_2/CO treatment

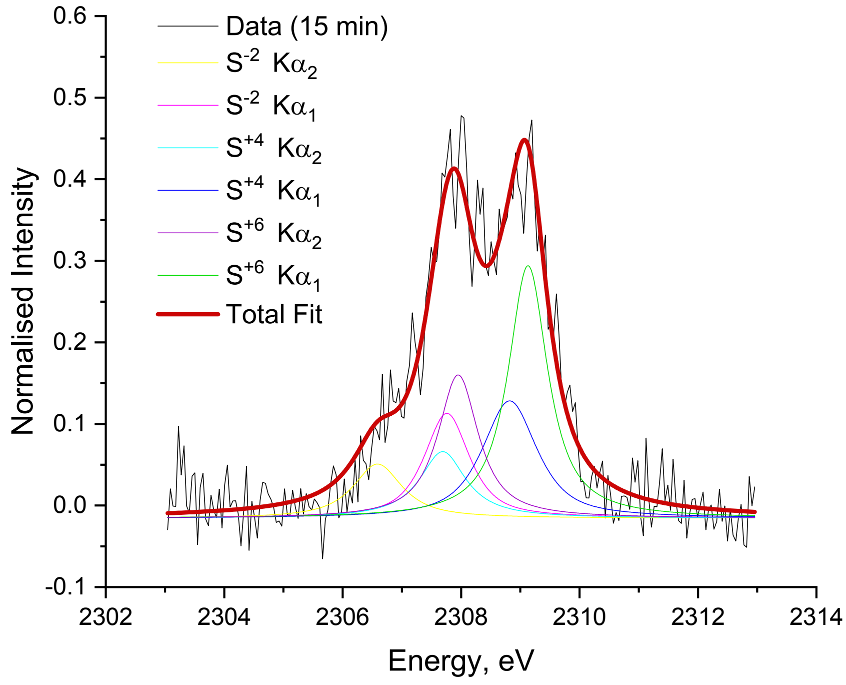

Fig. S4: 15 min into H_2/CO treatment

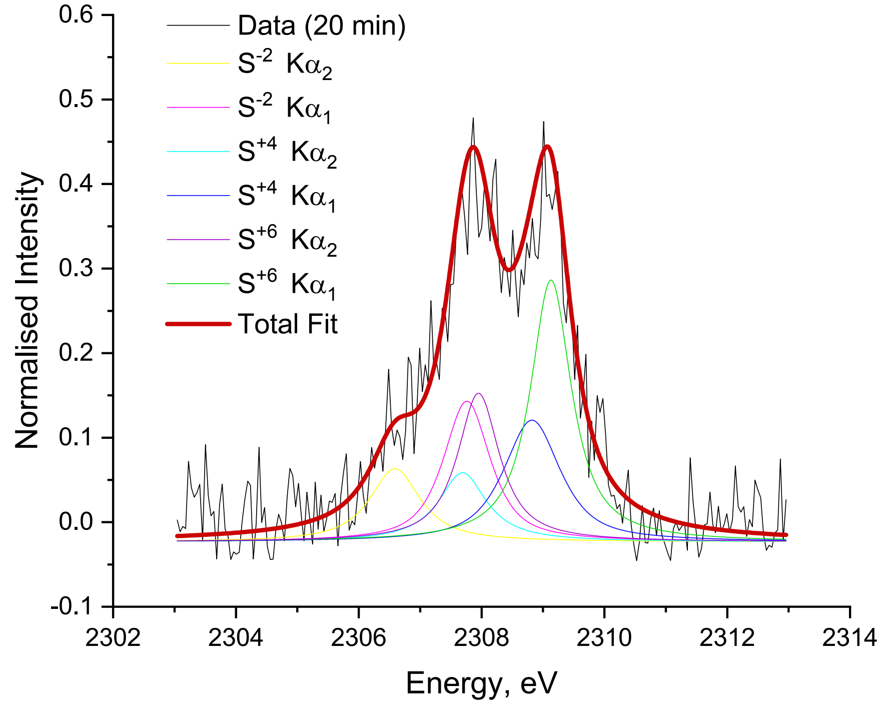

Fig. S5: 20 min into H_2/CO treatment

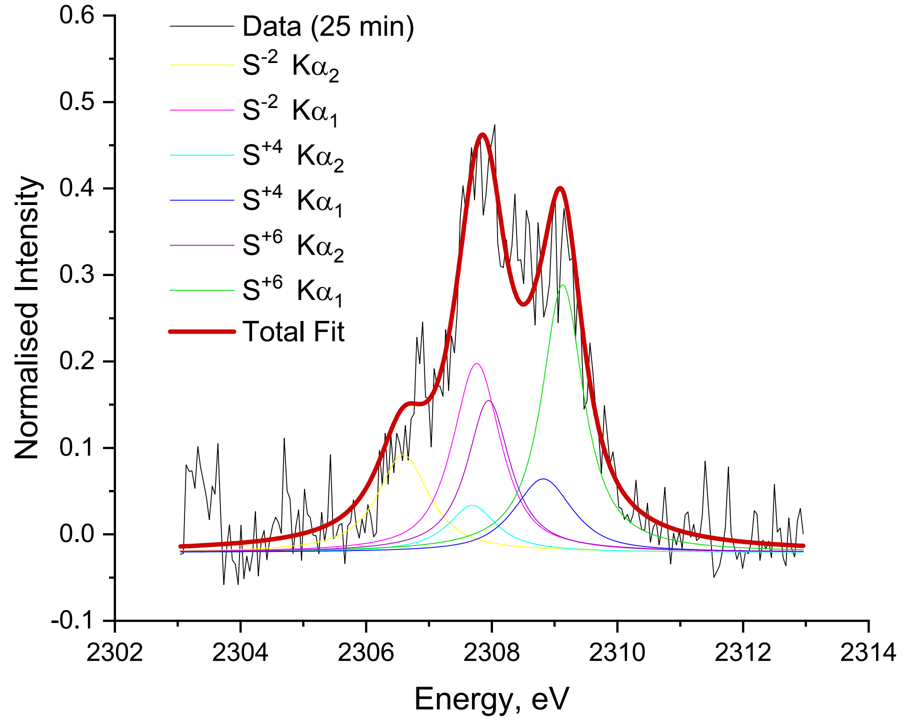

Fig. S6: 25 min into H_2/CO treatment

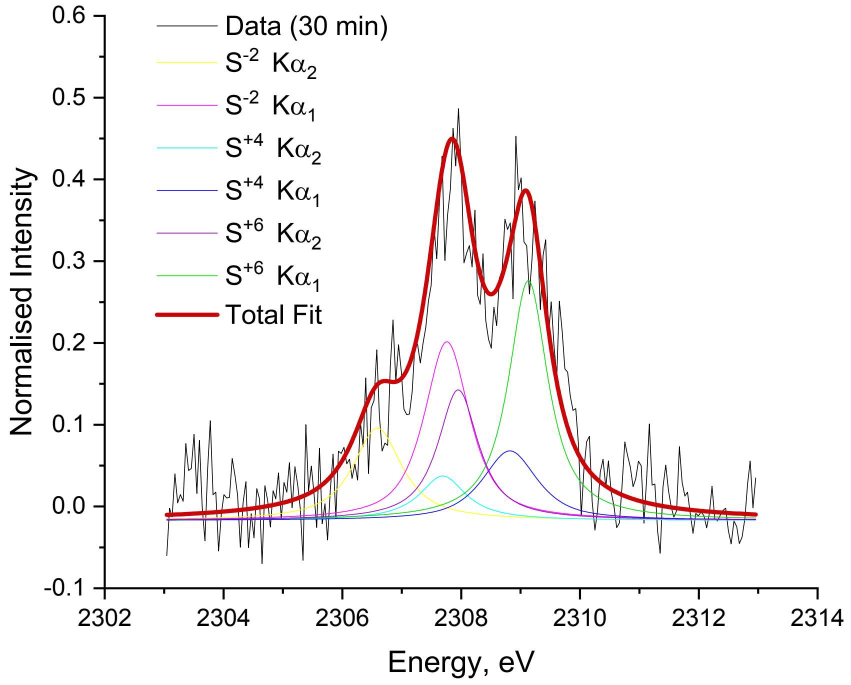

Fig. S7: 30 min into H_2/CO treatment

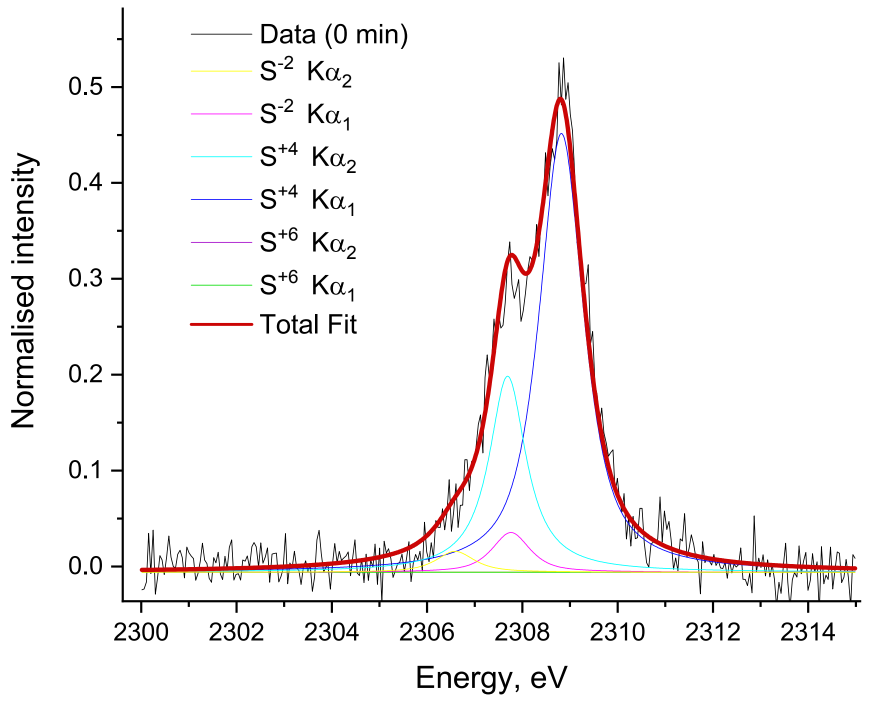

Fig. S8: 0 min into H_2/CO treatment

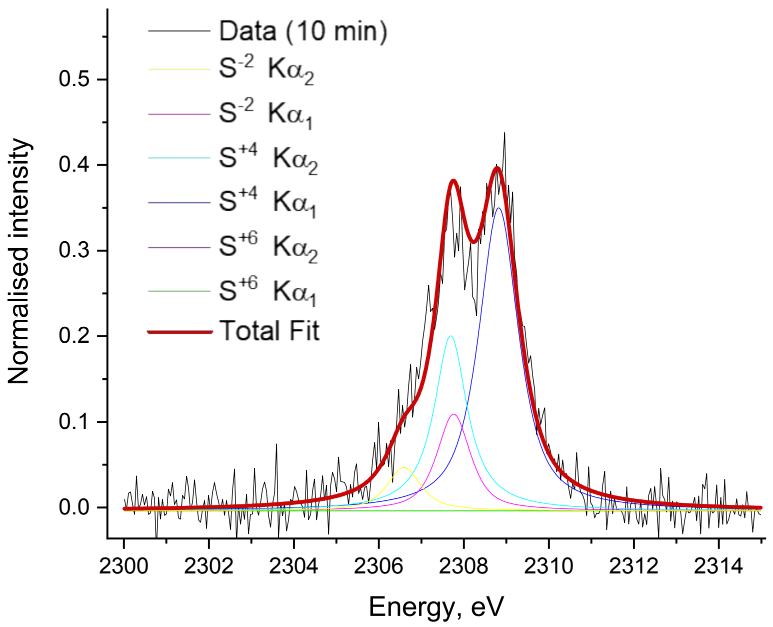

Fig. S9: 10 min into H_2/CO treatment

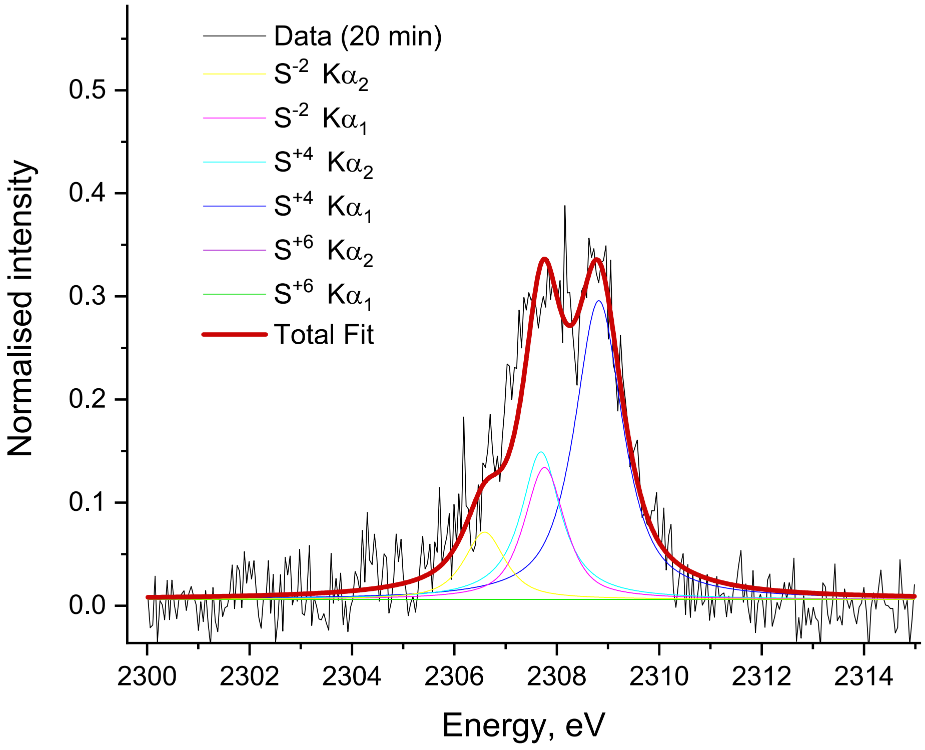

Fig. S10: 20 min into H_2/CO treatment

	$K\alpha_1$			$K\alpha_2$				
Reference	Center, eV	FWHM	mu	Center, eV	FWHM	mu	$K\alpha_1$/ $K\alpha_2$	A_{total}
S^{-2}	2307.76	0.91	0.83	2306.59	0.99	0.93	1.68	1.16
S^{+4}	2308.82	1.1	0.83	2307.69	0.94	0.99	1.82	1.07
S^{+6}	2309.13	0.8	0.95	2307.95	0.82	0.94	1.73	1.04

Table S1: Parameters used in fitting the in situ spectra

Time in H2/CO	% S^{-2}	% S^{+4}	% S^{+6}	Total area, a.u.	S^{-2} area, a.u.	S^{+4} area, a.u.	S^{+6} area, a.u.
0 min	9	39	52	3076431	276878.8	1199808.09	1599744.12
5 min	10.5	39	50.5	2197392	230726.2	856982.88	1109682.96
10 min	20.5	31	48.5	1369559	280759.6	424563.29	664236.115
15 min	22	29	49	977774	215110.3	283554.46	479109.26
20 min	27	27	46	828384	223663.7	223663.68	381056.64
25 min	36	17	47	641755	231031.8	109098.35	301624.85
30 min	37	18	45	695306	257263.2	125155.08	312887.7

Table S2: The percentage and area of each component in the fit during H_2/CO treatment after oxidative regeneration of Ru/Al_2O_3

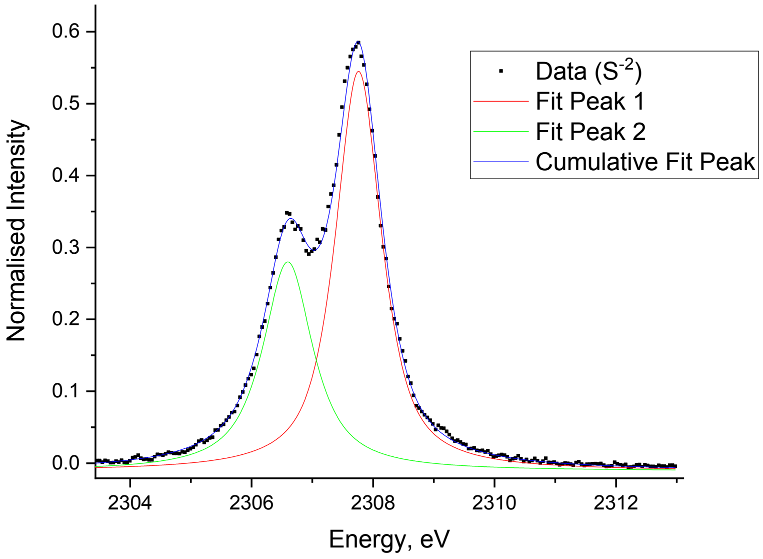

Fig. S11: Pseudo-Voigt fit of S^{-2} (Ag_2S) reference

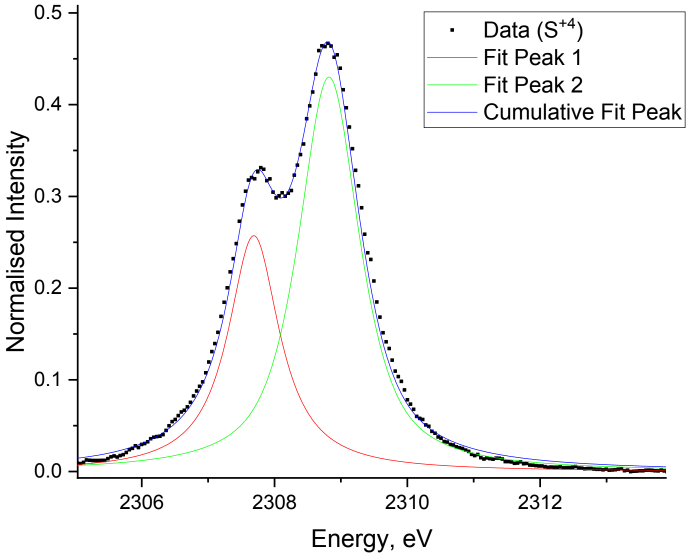

Fig. S12: Pseudo-Voigt fit of S^{+4} (Na_2SO_3) reference

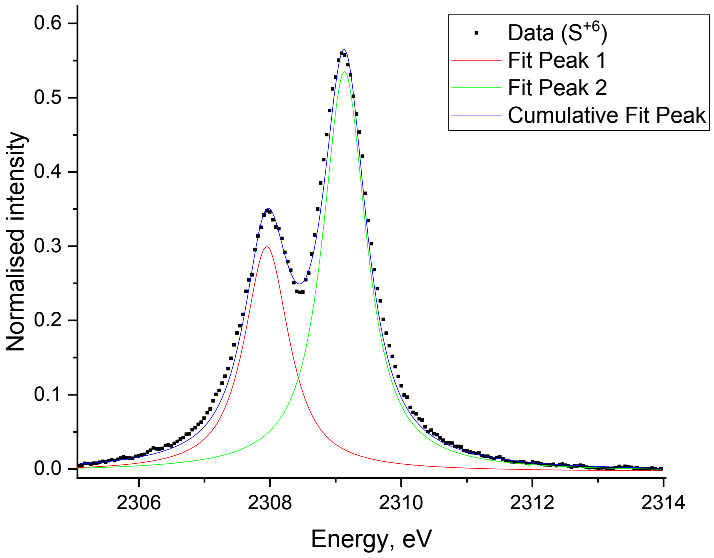

Fig. S13: Pseudo-Voigt fit of S^{+6} (Na_2SO_4) reference
